# Phage-delivered CRISPRi enables bacterial biocomputation

**DOI:** 10.1093/synbio/ysaf013

**Published:** 2025-07-20

**Authors:** Charlotte Ayn Cialek

**Affiliations:** Integrated DNA Technology, Inc., Coralville, IA 52241, United States

**Keywords:** biocomputation, microbe, CRISPRi, bacteriophage, microbial consortium

The information around us is increasingly being ingested and interpreted by computation, the process of applying logical operations to input data in order to generate the corresponding output. Beyond silicon-based devices, biological entities can also be programmed to serve as microscopic computers [1]. The emerging field of biocomputation aims to generate bio-based entities, including living cells, capable of serving specialized computational tasks. Living, growing microbes offer unique advantages over silicon-based devices. They self-repair, replicate, grow, and recover from damage, enabling signal processing in environments inaccessible to electronics. Microbes can detect, amplify, and report a signal when the cell recognizes a specific type of molecule, which enables biosensing, drug delivery, and environmental monitoring. Unlike electronics, microbes run on biochemical energy (ATP from metabolism, e.g.). Without requiring hardware to scale, self-replicating microbes can scale exponentially. By distributing distinct signals across separate cells, the resulting biomass allows for decentralized computation, with each cell functioning as a computing node.

However, the scalability of biocomputation remains limited by a lack of tools and methodologies. The few molecule-based circuits currently available restrict information capacity and require time-intensive, linearly scalable efforts to expand. Furthermore, advances in multi-cell circuits to build consortia of cells are likely needed to boost sensitivity and complexity and distribute the metabolic burden of biocomputational signalling [2]. To address these challenges, researchers at Université Paris-Saclay developed a scalable, CRISPR-based method enabling broad genomic targeting [3]. Paired with a novel bacteriophage delivery system, these circuits processed up to three coordinated inputs from distinct information-carrying cells. This work marks a significant step forward for synthetic biology, integrating bacteriophage-mediated CRISPR interference (CRISPRi) to enable multiplex biocomputation in microbial consortia.

Effective computation requires a system capable of sending, reading, and interpreting messages. In this work, sender cells transmitted messages of encoded guide RNAs that, upon delivery to receiver cells, could direct CRISPRi mechanisms to regulate expression of specific genes, a process termed intercellular CRISPR interference (i-CRISPRi). Thus, upon receiving a deactivated Cas9 guide RNA message, the receiver cell’s expression of that gene would be repressed. Because guide RNAs are genetically encoded, modifying their sequences allows i-CRISPRi to target virtually any gene for silencing. This programmable system of diverse signals is a significant step forward from many of the previous systems, which used a specific, unprogrammable signal molecule and therefore produced systems that were more constrained.

Importantly, for the circuit to perform correctly, the guide RNA messages must be effectively transmitted between the sender and receiver cells. This was accomplished by genetic information delivery via M13 bacteriophage, which infects bacteria with genetic material similar to how viruses infect human cells. Since M13 establish a non-lethal chronic infection, bacterial host cells can carry and transmit M13 with only a small impact to their metabolism and division. In nature, M13 bacteriophage uses the protein gp3φ to block reinfection. Harnessing this inherent capability, the researchers engineered two types of bacterial cells: the ‘senders’, gp3φ-positive cells only capable of transmitting a single message, and the ‘receivers’, gp3φ-negative cells capable of reinfection. The researchers could mix the sender cells and receiver cells together on different timescales and concentrations to understand the mechanism of message transmittance and optimize the system.

Once a 1-input system had been developed, the research team expanded to multiple input signals, allowing for increased biological circuit complexity. Additionally, the researchers expanded the ability of this system by also reversing the i-CRISPRi signal, by using de-repression instead of repression of gene expression. Using these advanced methodologies, the researchers built circuits with up to three input messages, and demonstrated the efficacy of seven different bacteriophage-carrying cell types. This bacteriophage-based communication system lays the groundwork for implementing more intricate biological circuits in engineered bacterial communities.

Biocomputation holds much promise to perform tasks that would be difficult or impossible for computers across a variety of applications. Naturally, microbes are constantly reading and interpreting chemical signals in their surrounding environments. By harnessing signal detection and writing genetic circuits, biocomputation could enable a monitoring system to quickly report molecular signals. For example, biocomputation could alert us to dangerous compounds like heavy metal contamination or explosives [4, 5], or our gut microbiome could be programmed to detect and diagnose certain maladies or pathogens [6–8]. Furthermore, microbes can be engineered to perform specialized functions, like catalysing reactions or releasing molecular cargo [9]. Thus, biocomputational signals could be harnessed to not only sense a problem but solve it, by triggering custom, specific outputs like producing biocontainment molecules or delivering specific drugs directly at the time and site of detection.

To realize such versatile future applications, biocomputing systems must overcome several challenges. One such challenge is that lab-optimized consortia often fail in real-world environments due to unpredictable microbial interactions, pH changes, or temperature fluctuations. The i-CRISPRi work demonstrated that bacteriophage-mediated communication efficiency depends on sender and receiver cell growth stages and competition for cellular resources. While communication was possible under optimized lab conditions, robust signal transmission in more complex environments, such as the human gut or natural substrate, will likely be challenging.

Another future hurdle will likely be expanding the signals beyond three distinct i-CRISPRi signals per cell. The study suggests that signal activation decreases with multiple message inputs, as single, dual, and triple inputs activate signals 21-fold, 14.3-fold, and 7.7-fold, respectively. Further optimization or increased sensitivity could enable circuits with more than three inputs without signal loss from growth or metabolic differences [10]. This step in optimizing the sensitivity of multi-cell consortia demonstrates the promise for a future where sophisticated, living biocomputers can detect, store, and report the molecular phenomena happening around—and perhaps even within—us.

## Data Availability

No datasets were generated or analysed during the current study.

## Notes

Conflict of interest: Charlotte Cialek is an employee of Integrated DNA Technologies (IDT) and co-owner of Firelight Biosciences, which both operate in the field discussed in this review. The author has no other relevant affiliations or financial involvement with any organization or entity with a financial interest in the subject matter of this review. The views expressed in this article are solely those of the author and do not necessarily represent the views of IDT or Firelight Biosciences.
